# Description of a new species of *Megischus* Brullé (Hymenoptera, Stephanidae), with a key to the species from China

**DOI:** 10.3897/zookeys.1022.62833

**Published:** 2021-03-08

**Authors:** Si-Xun Ge, Hong-Liang Shi, Li-Li Ren, Jiang-Li Tan

**Affiliations:** 1 College of Forestry, Beijing Forestry University, Beijing 100083, China Beijing Forestry University Beijing China; 2 Shaanxi Key Laboratory for Animal Conservation / Key Laboratory of Resource Biology and Biotechnology in Western China, College of Life Sciences, Northwest University, 229 North Taibai Road, Xi’ an, Shaanxi 710069, China Northwest University Xian China

**Keywords:** Distribution, largest Stephanidae, parasitoids, taxonomy, wasp

## Abstract

A new species of the genus *Megischus* Brullé, 1846, *Megischus kuafu* Ge & Tan, **sp. nov.**, is described and illustrated from Guizhou Province, China. The key to all four species from China is included. A distribution map of the Chinese species is added.

## Introduction

The small family Stephanidae Leach, 1815, consisting of 364 extant species, is cosmopolitan but mainly restricted to the subtropical and tropical areas (van Achterberg 2002; Aguiar 2004, 2006; Aguiar and Jennings 2005; van Achterberg and Quicke 2006; Aguiar et al. 2010; Hong et al. 2010, 2011; Tan et al. 2015a, b, 2018; Chen et al. 2016; Moghaddam et al. 2019; Binoy et al. 2020; Gupta and Gawas 2020). Species of Stephanidae are generally parasitoids of coleopterous larvae, including species of Buprestidae, Cerambycidae, and even Curculionidae, but also hymenopterous larvae of Siricidae (Chao 1964; Taylor 1967; Kirk 1975; Königsmann 1978; van Achterberg 2002; Aguiar 2004). The stephanids which are conspicuous by the five tubercles on the head (thus the name *stephanos*, Greek for crown), are considered to be rare and nearly 95% of all the species are described from a single specimen (Aguiar 2001; van Achterberg 2002). Among them, *Megischus* Brullé, 1846 is a large genus of Stephanidae with 87 species worldwide and 30 species from the Oriental region (van Achterberg 2002; van Achterberg and Yang 2004; Hong et al. 2010, 2011; Binoy et al. 2020). However, there are only three species known from China up to date (Hong et al. 2010, 2011). *Megischus* contains the largest known species of Stephanidae with a body length up to 35 mm, excluding the ovipositor (Hong et al. 2011). Here we report the fourth species of the genus from the Oriental part of China with a body length of 39 mm.

## Materials and methods

The holotype was collected by sweep net and directly preserved in 70% alcohol. For identification of the family Stephanidae and genera, van Achterberg (2002) and Hong et al. (2011) were used.

The descriptions, measurements, and figures were made using a Leica M205A microscope with a Leica Microsystem DFC550 digital camera. Photographs were combined using the Leica Application Suite (Version 4.5.0). Morphological nomenclature follows van Achterberg (2002) including the abbreviations for the wing venation. The holotype is deposited in the College of Forest Protection, Beijing Forestry University (**BFU**), China.

## Taxonomy

### Genus Megischus

Taxon classificationAnimaliaHymenopteraStephanidae

Brullé, 1846

C730325B-039C-5BC1-B250-65DC2E1C9C47

Megischus Brullé, 1846: 537. Type species (designated by Viereck 1914): M. annulator Brullé, 1846 [= M. furcatus (Lepeletier & Serville, 1825)].
Megischus Brullé, 1846: van Achterberg 2002: 53–168; Aguiar and Johnson 2003: 469–482.
Bothriocerus Sichel, 1860: 759. Type species: Bothriocerus europaeus Sichel, 1860 (by monotypy) (= Stephanus anomalipes Foerster, 1855, according to Madl 1991).

#### Diagnosis.

Medium to large size. Temple without pale yellowish streak behind eye. Pronotum robust without transverse protuberance. First subdiscal cell of fore wing comparatively narrow basally, approximately as wide as first discal cell or narrower; vein 1-SR of fore wing differentiated with first discal cell present because of presence of vein 1-SR+M; vein 1-M and vein 2-SR straight or nearly so. Hind wing without trace of vein cu-a. Hind coxa without dorsal tooth; hind femur with two distinct teeth; hind tibia narrowed basally and inner side usually with wide sub-medical depression, evenly rounded ventrally and without oblique striae or rugae on the outer sides; hind tarsus with three tarsomeres. Sternite I not differentiated from tergite I. Tergite I 4.2–17.6 × as long as its apical width, cylindrical, distinctly longer than tergite II; tergite II more or less petiolate and sculptured basally. Ovipositor sheath with ivory subapical band.

#### Distribution.

Cosmopolitan. The distribution of Chinese species is illustrated in Fig. 20.

#### Note.

*Megischus* specimens are still poorly collected. The known diversity in China compared with the diversity outside China is low and higher numbers of species can be expected.

### Key to Chinese species of the genus *Megischus* Brullé

**Table:** 

1	Head orange brown, temple distinctly convex behind eye; neck rather short and robust, anteriorly rather shallowly concave; middle pronotum steeply rises from neck postero-dorsally; vein 1-M of fore wing ca 2.2 × as long as vein 1-SR; widest part of hind tibia of male nearly straight ventrally. [Pronotal fold absent; vein 1-M of fore wing 0.9 × vein m-cu; hind basitarsus ca 3.5 × as long as wide. Female unknown] (Hubei)	***M. aplicatus* Hong, van Achterberg & Xu, 2010**
–	Head dark brown or reddish brown, temple slightly convex or narrowed behind eye; neck elongate and anteriorly distinctly concave (in some specimens of *M. ptosimae* shallowly emarginate); neck at same or lower level than middle part of pronotum postero-dorsally; vein 1-M of fore wing more than 4 × as long as vein 1-SR; widest part of hind tibia weakly to distinctly concave ventrally.	**2**
2	Head brown, temple narrowly rounded medially behind eye in dorsal view; pronotal fold and concavity absent; medially middle part of pronotum at same level with posterior part postero-dorsally. [vein 1-M of fore wing ca 5.0 × as long as vein 1-SR and 1.2 × vein m-cu.] (Fujian)	***M. chaoi* van Achterberg, 2004**
–	Head dark brown or reddish brown, temple slightly convex behind eye in dorsal view; pronotal fold distinct and with a cavity below it; neck at lower level than middle pronotum postero-dorsally. [vein 1-M of fore wing ca 4.2–5.9 × as long as vein 1-SR and 0.7–1.3 × vein m-cu]	**3**
3	Head dark brown and malar space pale yellowish; vein 1-M of fore wing ca 4.2–5.5 × as long as vein 1-SR and 1.1–1.3 × vein m-cu; widest part of hind tibia distinctly concave ventrally; hind basitarsus 3.0–3.5 × as long as wide; ivory part of ovipositor sheath 0.7–2.0 × as long as dark apical part (Guangdong, Shaanxi, Sichuan, Zhejiang, Fujian)	***M. ptosimae* Chao, 1964**
–	Head completely dark reddish brown (red in alive specimen; Fig. 19); vein 1-M of fore wing ca 5.9 × as long as vein 1-SR and 0.8 × vein m-cu; widest part of hind tibia weakly concave ventrally; hind basitarsus ca 7.4 × as long as wide; ivory part of ovipositor sheath ca 2.0 × as long as dark apical part (Guizhou)	***M. kuafu* Ge & Tan, sp. nov.**

### Megischus kuafu

Taxon classificationAnimaliaHymenopteraStephanidae

Ge & Tan sp. nov.

E7E05FA6-A9DF-5709-B8B4-B53C45786FDB

http://zoobank.org/3CDF81C0-D859-45F8-8E57-E3A77CBC9615

Figures 1–5 , 6–8 , 9–12 , 13–16 , 17–19

#### Material examined.

***Holotype***, ♀ (BFU), China: Guizhou, Libo, Maolan National Nature Reserve; Wuyanqiao; 108°6.065'E, 25°17.598'N, 541 m, 26.V.2020, leg. Si-Xun Ge.

#### Diagnosis.

Head completely dark reddish brown (red in alive specimen; Fig. 19), temples slightly bulging behind eyes; ocellar area (Fig. 2) transversely rugose; vertex reticulate-rugose medially, followed by weakly transverse rugae posteriorly almost reaching occipital carina; pronotum (Fig. 4) subparallel anteriorly and with distinct pronotal fold; apical median portion of neck shiny (before protonal fold); medio-anterior pronotum moderately wide (in dorsal view) and strong transverse rugae; scutellum (Fig. 6) almost glabrous and with foveolae laterally; vein 1-M ca 5.9 × as long as vein 1-SR; hind basitarsus densely setose and parallel-sided, ventral length 7.4 × maximum width.

**Figures 1–5.**
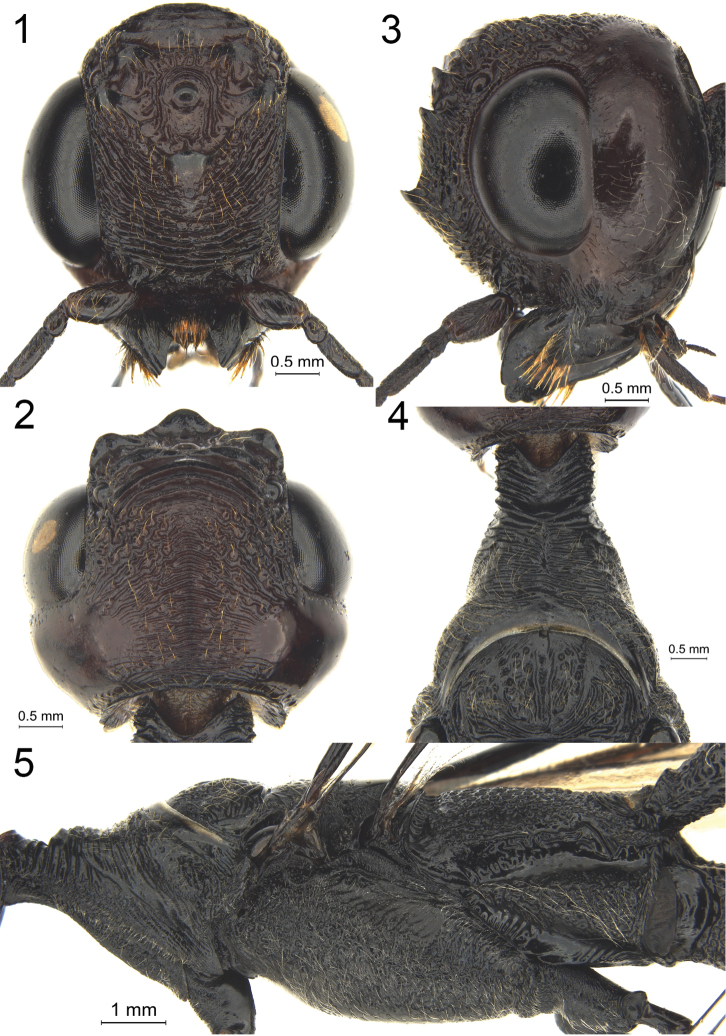
*Megischus kuafu* Ge & Tan, sp. nov. Holotype ♀ **1** head, frontal view **2** head, dorsal view **3** head, lateral view **4** pronotum, dorsal view **5** mesosoma, lateral view.

#### Description.

***Holotype*. Female.** Length of body 39.1 mm; forewing 21.3 mm; ovipositor sheath 59 mm.

***Head*.** Antenna with 39 flagellomeres; the first flagellomere slender, length 3.4 × its maximum width, and length of second flagellomere 1.2 × its width; frons coarsely and transversely rugose (Fig. 1); three anterior coronal teeth large and lobe-shaped, both posterior ones smaller and wider; vertex transversely rugose anteriorly and reticulate-rugose medially, followed by coarsely and slightly curved rugosities reaching occipital carina; temple slightly bulging, smooth and shiny (Fig. 2), except for some fine punctures laterally; occipital carina strongly developed and connected to hypostomal carina; hypostomal carina large and without distinct rugae, only some punctures (Fig. 3).

**Figures 6–8.**
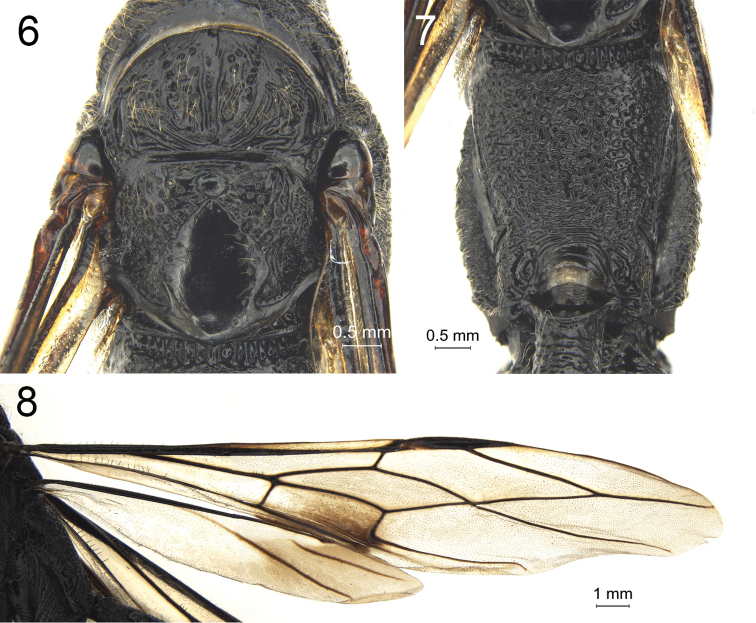
*Megischus kuafu* Ge & Tan, sp. nov. Holotype ♀ **6** mesoscutum and scutellum, dorsal view **7** propodeum, dorsal view **8** wings.

***Mesosoma*.** Neck robust and anteriorly distinctly concave (Fig. 4), with several weak incomplete carina anteriorly and three interrupted and rather strong carina, at lower level than middle part of pronotum postero-dorsally (Fig. 5), and with large smooth and shiny area before pronotal fold; pronotal fold strong, weakly sinuate and below it with rather deep concavity (Fig. 4); middle part of pronotum with nine weak and irregular transverse carinae (as laterally) and with distinct oblique lateral groove; no median carina anteriorly; middle part of pronotum weakly differentiated from posterior part (Fig. 5), and latero-posteriorly rather weakly convex; posterior part of pronotum generally with rather sparse setosity, latero-ventrally densely setose but dorso-posteriorly glabrous, with several coarse punctures and latero-posteriorly with some crenulae; propleuron coriaceous and setose; prosternum densely foveolate, foveolae circular and setose; convex part of mesopleuron strongly foveolate and with dense short whitish setosity (Fig. 5); mesosternum largely smooth (except some fine punctures); scutellum smooth and with foveolae laterally (Fig. 6); propodeum dorsally almost glabrous (Fig. 7), completely with shallow, circular foveolae, most foveolae are separated and some of them coalescent.

***Wings*.** Fore wing: wing membrane largely subhyaline (Fig. 8), and surface evenly bristly; vein M+CU1 with four short, erect, equidistant spiny setae; vein 1-M 5.9 × as long as vein 1-SR and 0.8 × vein m-cu; vein 2-SR 0.9 × as long as vein r; vein r ends 0.5 × length of pterostigma behind the level of apex of pterostigma; vein 1-SR 1.1 × as long as parastigmal vein; vein 3-CU1 distinct and curved apically.

**Figures 9–12.**
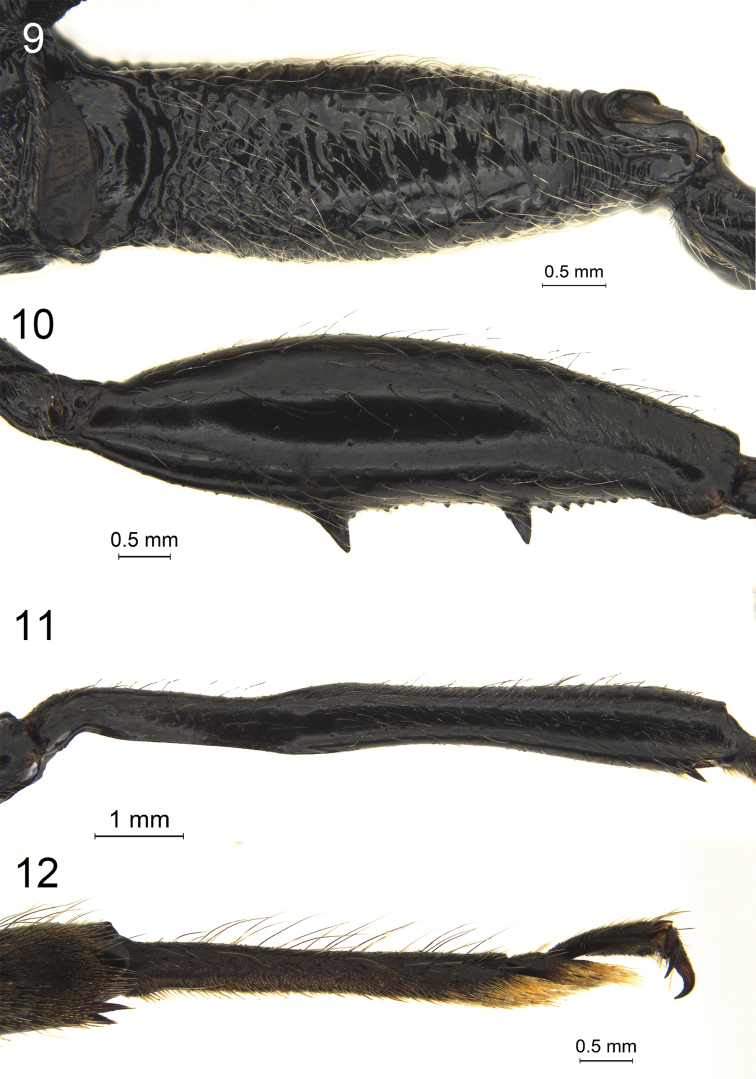
*Megischus kuafu* Ge & Tan, sp. nov. Holotype ♀ **9** hind coxa, lateral view **10** hind femur, lateral view **11** hind tibia, lateral view **12** hind tarsi, lateral view.

***Legs*.** Hind coxa rather strong, annular, largely transversely striate, with long whitish setosity strongly inclined towards (Fig. 9); hind femur robust, with scattered punctures and largely smooth and shiny interspaces (Fig. 10), hind femur ventrally with two large teeth and ten minute teeth in between and one small tooth behind large posterior tooth; hind tibia distinctly curved basally (Fig. 11), elongate and 1.2 × longer than hind femur, densely setose and mostly sparsely punctate, basal narrow part of hind tibia 0.5 × as wide as widest part, lateral view of hind tibia below depression nearly parallel-sided and slender, inner side rather convex basally, densely setose; hind basitarsus slender and parallel-sided, bristly setose ventrally, ventral length 7.4 × its maximum width (Fig. 12).

**Figures 13–16.**
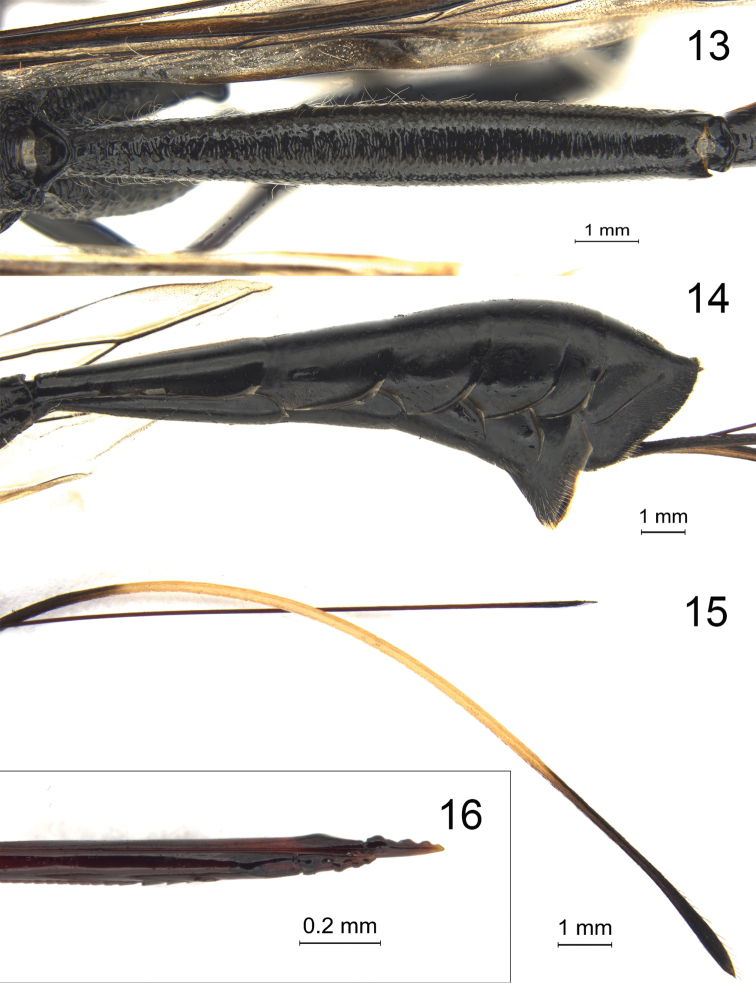
*Megischus kuafu* Ge & Tan, sp. nov. Holotype ♀ **13** tergite I, dorsal view **14** metasoma (except tergite I), lateral view **15** distal part of ovipositor and sheath, lateral view **16** apex of ovipositor, lateral view.

***Metasoma*.** Tergite I transversely striate-rugose (Fig. 13), ca 6.9 × as long as its maximum width and 10.4 × its apical width, 1.9 × as tergite II and 0.7 × as remainder of metasoma; basal 0.1 of tergite II rugose, remainder smooth and glabrous; remainder of tergites (Fig. 14) shiny and with sparse and short setae (except tergite VII densely setose medially); pygidial area coriaceous, medially moderately convex and distinctly punctate medially and anteriorly, with long straight setae; length of ovipositor sheath ca 1.5 × as long as body and ca 2.8 × as long as forewing, length of subapical whitish band (Fig. 15) twice as long as dark apical part. Ovipositor tip laterally compressed, with minute teeth apically (Fig. 16).

***Colour*.** Mostly black; mesosoma, metasoma, antennae, and hind legs black or blackish; head dark reddish brown; tergite II brownish bilaterally; wing membrane light brownish, hyaline, except most of hind portion of first subdiscal cell and apical part of hind wing brown; veins and pterostigma brown or dark brown; fore and middle legs dark brown (except for coxae black); ovipositor sheath largely black and with whitish subapical band.

**Male.** Unknown.

#### Etymology.

The species name is derived from the name of a giant chasing the sun in Chinese mythology, as an analogy of its exclusively large size and a dark reddish-brown head.

#### Distribution.

China (Guizhou).

#### Biology.

Collected in May. Host is unknown.

#### Note.

The description is based on the pinned holotype. The colour of the head changed from bright red into dark reddish brown after it died (Fig. 19). The genus *Megischus* contains the largest known stephanids and some of them can be up to 35 mm (Binoy et al. 2020). Although the size of parasitoids varies among specimens of the same species due to the nutritional conditions of the host and other factors, the body length of 39 mm makes *M. kuafu* the largest known *Megischus* specimen, and also the largest Stephanidae.

**Figures 17–19.**
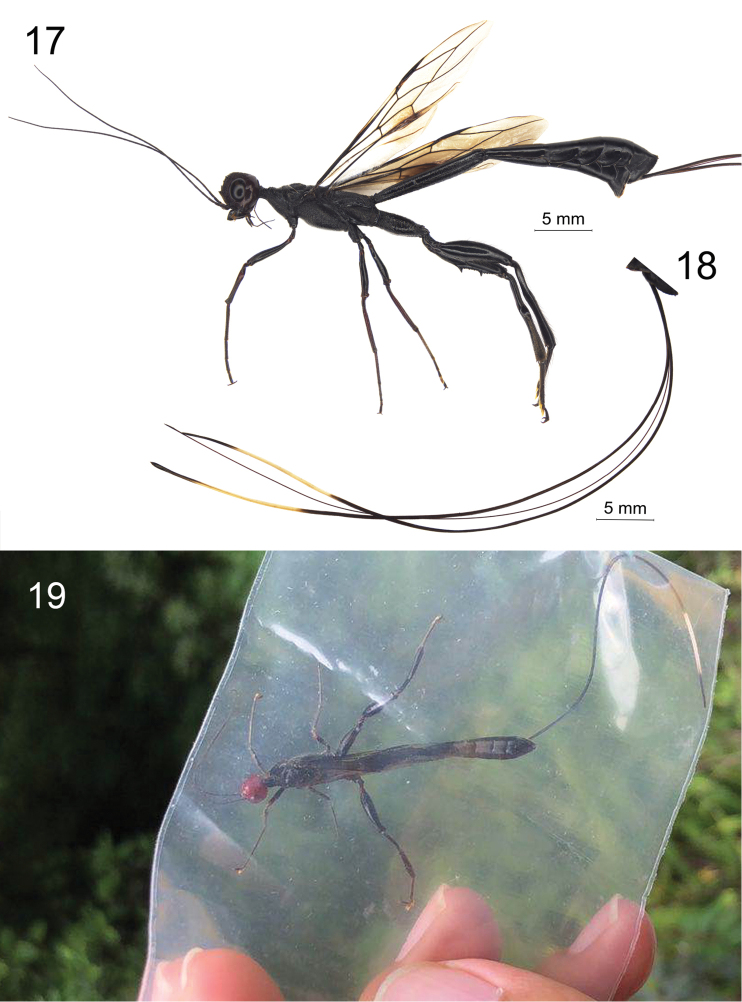
**17** habitus of holotype. ♀ *Megischus kuafu* Ge & Tan, sp. nov. (except ovipositor and ovipositor sheath) **18** ovipositor and ovipositor sheath **19** collecting living specimen.

The large size and general colour pattern more or less resemble *M. ducaloides* van Achterberg, 2004, but it can be easily distinguished from it by the distinct pronotal fold and the rounded shape of the posterior part of the pronotum. The new species runs to *M. ptosimae* in the key to Chinese species by Hong et al. (2011) in having the temple slightly convex behind eye, a distinct pronotal fold and cavity below it, and vein 1-M of fore wing ca 5.5 × as long as vein 1-SR. However, the new species differs from *M. ptosimae* in lacking a pale yellowish malar space, vein 1-M 0.8 × as long as vein m-cu of the fore wing, less sculptured scutellum, posterior half of the hind tibia weakly concave ventrally and the hind basitarsus ca 7.4 × as long as wide. This new species runs to *M. rubripes* (Kieffer, 1916) in the key to Old World *Megischus* by van Achterberg (2002), but it differs from *M. rubripes* in having a more irregular sculpture of the vertex, a large, smooth, and shiny concavity before the pronotal fold, blackish hind tibia and hind basitarsus and tergite I ca 6.9 × as long as its maximum width.

**Figure 20.**
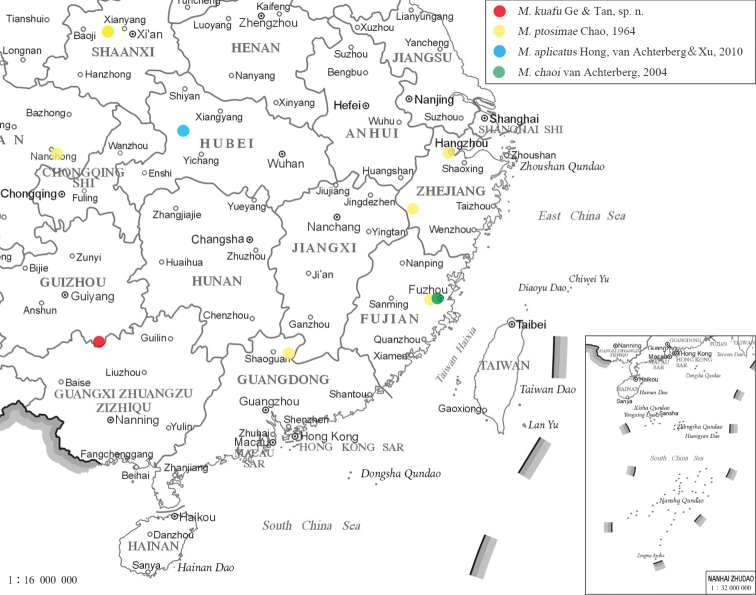
Distribution map of *Megischus* species from China (map of China from: http://bzdt.ch.mnr.gov.cn/).

## Supplementary Material

XML Treatment for Megischus

XML Treatment for Megischus kuafu
